# Associations of maternal early-pregnancy blood glucose and insulin concentrations with DNA methylation in newborns

**DOI:** 10.1186/s13148-020-00924-3

**Published:** 2020-09-07

**Authors:** Madelon L. Geurtsen, Vincent W. V. Jaddoe, Romy Gaillard, Janine F. Felix

**Affiliations:** 1 The Generation R Study Group, Erasmus MC, University Medical Center Rotterdam, PO Box 2040, 3000 CA Rotterdam, The Netherlands; 2 Department of Pediatrics, Erasmus MC, University Medical Center Rotterdam, Rotterdam, The Netherlands

**Keywords:** Maternal glucose, Maternal hyperglycemia, Gestational diabetes, Maternal insulin, Diabetes mellitus, DNA methylation, Epigenetics, Differentially methylated regions

## Abstract

**Background:**

Intrauterine exposure to a disturbed maternal glucose metabolism is associated with adverse offspring outcomes. DNA methylation is a potential mechanism underlying these associations. We examined whether maternal early-pregnancy glucose and insulin concentrations are associated with newborn DNA methylation. In a population-based prospective cohort study among 935 pregnant women, maternal plasma concentrations of non-fasting glucose and insulin were measured at a median of 13.1 weeks of gestation (95% range 9.4–17.4). DNA methylation was measured using the Infinium HumanMethylation450 BeadChip (Ilumina). We analyzed associations of maternal early-pregnancy glucose and insulin concentrations with single-CpG DNA methylation using robust linear regression models. Differentially methylated regions were analyzed using the dmrff package in R. We stratified the analyses on normal weight versus overweight or obese women. We also performed a look-up of CpGs and differently methylated regions from previous studies to be associated with maternal gestational diabetes, hyperglycemia or hyperinsulinemia, or with type 2 diabetes in adults.

**Results:**

Maternal early-pregnancy glucose and insulin concentrations were not associated with DNA methylation at single CpGs nor with differentially methylated regions in the total group. In analyses stratified on maternal BMI, maternal early-pregnancy glucose concentrations were associated with DNA methylation at one CpG (cg03617420, *XKR6*) among normal weight women and at another (cg12081946, *IL17D*) among overweight or obese women. No stratum-specific associations were found for maternal early-pregnancy insulin concentrations. The two CpGs were not associated with birth weight or childhood glycemic measures (*p* values > 0.1). Maternal early-pregnancy insulin concentrations were associated with one CpG known to be related to adult type 2 diabetes. Enrichment among nominally significant findings in our maternal early-pregnancy glucose concentrations was found for CpGs identified in a previous study on adult type 2 diabetes.

**Conclusions:**

Maternal early-pregnancy glucose concentrations, but not insulin concentrations, were associated with DNA methylation at one CpG each in the subgroups of normal weight and of overweight or obese women. No associations were present in the full group. The role of these CpGs in mechanisms underlying offspring health outcomes needs further study. Future studies should replicate our results in larger samples with early-pregnancy information on maternal fasting glucose metabolism.

## Background

The prevalence of gestational diabetes is rising worldwide and has been reported to complicate up to 25% of all pregnancies [1, 2]. This rise is partly due to the increasing prevalence of obesity among women of reproductive age and depends on screening tools and diagnostic criteria [2–4]. Intrauterine exposure to maternal gestational diabetes or impaired glucose tolerance measured in mid-pregnancy and late pregnancy is associated with increased risks of adverse maternal and fetal perinatal outcomes and of diabetes and obesity in the offspring [5–8]. These associations of increased risks on perinatal outcomes are already present for higher maternal glucose concentrations below the threshold of gestational diabetes [9, 10]. Additionally, the associations are stronger among women who are overweight or obese at the start of their pregnancy [2, 3]. Women who develop hyperglycemia and gestational diabetes may already have suboptimal glucose metabolism earlier in pregnancy. The first trimester of pregnancy is a critical period for embryonic and placental growth and development [11]. As such, impaired maternal glucose metabolism may already exert negative effects in that early stage. Early-pregnancy glucose metabolism has been shown to be associated with altered fetal growth, adverse birth outcomes, and childhood glucose metabolism, but not with other childhood cardiometabolic outcomes after adjusting for maternal pre-pregnancy BMI [9, 12, 13]. Thus, early pregnancy may be an important time window for the effects of suboptimal maternal glycemic measures and as such an influential period for future interventions.

The mechanisms underlying these associations are unknown. DNA methylation has been suggested as a potential mechanism linking adverse exposures during pregnancy and impaired offspring health [14, 15]. Previous studies using candidate-gene approaches suggested that maternal gestational diabetes is associated with epigenetic modifications in placenta and cord blood at loci relevant to growth, energy homeostasis, and diabetes mellitus [14, 16–18]. Epigenome-wide association studies (EWAS) of gestational diabetes or maternal glucose concentrations showed varying results, with no clear pattern of associations [1, 15, 19–25]. The inconsistent results of candidate-gene studies and EWAS may be due to differences in study design. The studies varied in their exposure definition: gestational diabetes as binary exposure or glucose concentrations after an oral challenge test, in the tissues in which DNA methylation was measured: placenta or blood, and in the extent of adjustment for covariates, with most not adjusting for cell-type heterogeneity. Also, the majority had limited sample sizes [15, 19, 21–25]. It is not known whether maternal glucose and insulin concentrations across the full range in early pregnancy are associated with cord blood DNA methylation and whether these associations differ between normal weight versus overweight or obese women. Insight into these associations and their underlying mechanisms is important, as maternal blood glucose metabolism can be a target for preventive interventions to improve child health outcomes.

We hypothesized that maternal early-pregnancy glucose and insulin concentrations are associated with offspring DNA methylation at birth. Therefore, we conducted an epigenome-wide association analysis in a population-based prospective cohort study, with maternal glucose and insulin concentrations measured at a median of 13.1 weeks of gestation (95% range 9.4–17.4). As a secondary analysis, we stratified on maternal body mass index (BMI) categories to observe if maternal BMI modifies the studied associations. We also examined whether differential DNA methylation at any CpGs found to be associated with maternal glucose or insulin concentrations in cord blood persisted in peripheral blood of 10-year-old children. To obtain further insight into the potential significance of the observed DNA methylation changes, we conducted exploratory analyses on the associations of identified CpGs with offspring health outcomes. We also performed a look-up in our results of CpGs identified to be associated with maternal glucose metabolism during pregnancy or with type 2 diabetes in adults in previous literature.

## Results

### Subject characteristics

The population characteristics for the total group and stratified on maternal BMI in women with normal weight versus women with overweight or obesity are shown in Table 1. The mean maternal early-pregnancy glucose concentration was 4.4 mmol/l (standard deviation 0.8) and the median maternal early-pregnancy insulin concentration was 126.3 pmol/l (95% range 19.9–774.6). Gestational diabetes was diagnosed in 12 (1.3%) women and 59 (6.3%) women were obese. Non-response analyses showed that mothers without data on early-pregnancy glucose and insulin measurements delivered more often female children (Additional file 1: Table S1).

**Table 1:** Maternal and birth characteristics of the study population

Characteristics	Total group, *n* = 935	Maternal normal weight, *n* = 667	Maternal overweight/obesity, *n* = 234	*p* value
Maternal characteristics				
Age, years	31.7 ± 4.2	31.8 ± 4.2	31.7 ± 4.1	0.69
Height, cm	170.8 ± 6.2	171.1 ± 6.3	170.4 ± 5.8	0.25
Pre-pregnancy body mass index, kg/m^2^	23.3 ± 3.8	21.8 ± 1.6	28.5 ± 3.4	< 0.01
Women with				
Underweight	34 (3.6)	–	–	
Normal weight	667 (71.3)	667	–	
Overweight	175 (18.7)	–	175	
Obesity	59 (6.3)	–	59	
Gestational age at glucose/insulin measurement, weeks	13.1 (9.4–17.4)	13.4 (8.3–17.4)	12.9 (9.5–17.5)	0.19
Parity, nulliparous	563 (60.3)	409 (61.3)	130 (55.6)	0.23
Education, higher education	596 (64.9)	405 (71.6)	97 (50.0)	< 0.01
Smoking during pregnancy, continued	173 (20.8)	101 (19.6)	40 (22.6)	0.40
Glucose, mmol/l	4.4 ± 0.8	4.4 ± 0.8	4.5 ± 0.8	0.08
Insulin, pmol/l	126.3 (19.9–774.6)	119.6 (19.6–764.5)	153.6 (19.0–847.4)	0.09
Gestational diabetes	10 (1.3)	8 (1.7)	1 (0.5)	0.31
Child characteristics				
Male	491 (52.5)	311 (54.4)	96 (49.0)	0.19
Gestational age at birth, weeks	40.3 (36.4–42.3)	40.4 (36.5–42.3)	40.4 (36.3–42.3)	0.60
Birth weight, grams	3552 ± 514	3552 ± 493	3599 ± 571	0.31
Glucose, mmol/l	5.2 (0.9)	5.2 (0.9)	5.0 (0.9)	0.03

Values are means ± SD, medians (95% range) or numbers of subjects (valid %) shown for the total group and stratified for maternal pre-pregnancy body mass index. The stratified groups are normal weight versus overweight or obese women (data for underweight women are not separately shown *n* = 34). Differences were tested using Student’s *t* tests and Mann-Whitney tests for normally and non-normally distributed variables, respectively, and *χ*2 test were used for dichotomous variables

### Associations of maternal early-pregnancy glucose and insulin concentrations with DNA methylation at birth

After Bonferroni (*p* value cutoff < 1.0 × 10^−7^) or false-discovery rate (FDR) correction, we did not observe associations of maternal early-pregnancy glucose or insulin concentrations with offspring DNA methylation in cord blood. These models were adjusted for gestational age at glucose/insulin measurement, maternal age, educational level, parity, smoking, pre-pregnancy BMI, child sex, cell-type proportions, and batch. The results of both analyses are presented in Additional file 2: Figure S1a and Figure S1b. The CpGs with *p* values < 1.0 × 10^−4^ for both analyses are shown in Additional file 3: Table S2 and Table S3. A model without adjustment for maternal pre-pregnancy BMI showed largely similar results (Additional file 4: Table S4 and Table S5).

**Fig. 1:**
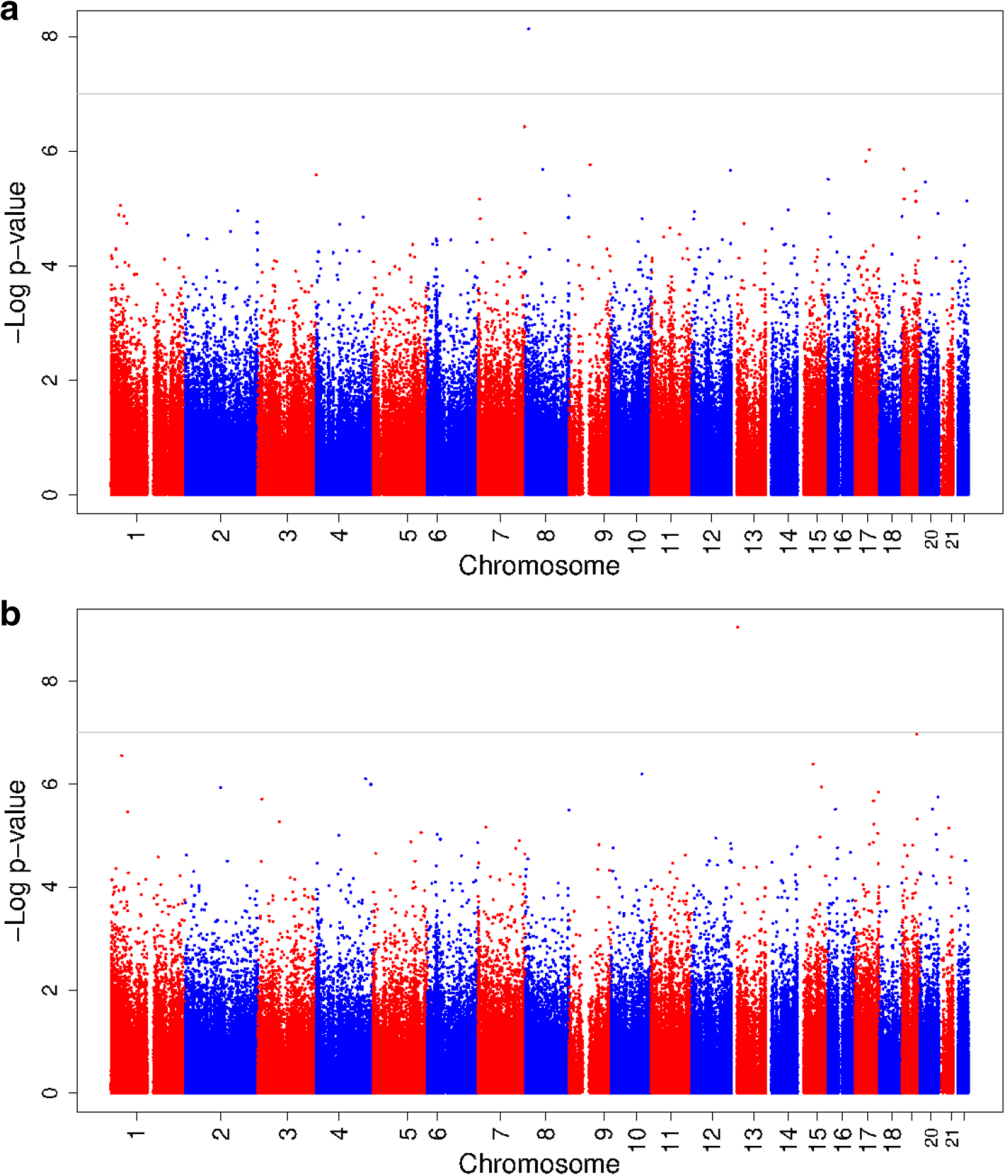
**a** Epigenome-wide association study results of maternal early-pregnancy glucose concentrations and DNA methylation in cord blood in normal weight women. **b** Epigenome-wide association study results of maternal early-pregnancy glucose concentrations and DNA methylation in cord blood in overweight or obese women. In **a**, the Manhattan plot shows the results of the epigenome-wide association study of maternal early-pregnancy glucose concentrations and DNA methylation in cord blood in normal weight women. In **b**, Manhattan plot of the results of the epigenome-wide association study of maternal early-pregnancy glucose concentrations and DNA methylation in cord blood in overweight or obese women. In both figures, the *x*-axis represents the autosomal (1–22) chromosomes and the *y*-axis shows the –log_10_ (*p* value). The models were adjusted for gestational age at assessment, maternal age at intake, educational level, parity, smoking, child sex, cell type proportions, and batch

The analyses stratified on maternal BMI showed that among normal weight women, maternal early-pregnancy glucose concentrations were associated with DNA methylation at one CpG (cg03617420 in *XKR6*; effect estimate = 7.3 × 10^−3^ (standard error (SE) 1.3 × 10^−3^), *p* value = 7.4 × 10^−9^) (Fig. 1a). DNA methylation at this CpG was not significantly associated with glucose concentrations in overweight or obese women (effect estimate = − 2.4 × 10^−3^ (SE 3.2 × 10^−3^), *p* value = 0.46). Among overweight or obese women maternal early-pregnancy glucose concentrations were associated with DNA methylation at one CpG (cg12081946 in *IL17D*; effect estimate = − 3.4 × 10^−2^ (SE 5.6 × 10^−3^), *p* value = 8.9 × 10^−10^) (Fig. 1b). DNA methylation at this CpG was not significantly associated with glucose concentrations in normal weight women (effect estimate = 3.1 × 10^−3^ (SE 4.1 × 10^−3^), *p* value = 0.45). Maternal early-pregnancy insulin concentrations were not associated with DNA methylation in cord blood in normal weight and in overweight or obese women (Additional file 5: Figure S2a and Figure S2b).

Neither maternal early-pregnancy glucose nor insulin concentrations were associated with differentially methylated regions in cord blood, analyzed using the dmrff package in R [26]. The differentially methylated regions with *p* values < 1.0 × 10^−4^ are shown in Additional file 6: Table S6 and Table S7.

Maternal glucose concentrations were not associated with DNA methylation levels at the two identified CpGs in peripheral blood of 10-year-old children (*p* values > 0.1) (Additional file 7: Table S8). In exploratory analyses, the two identified CpGs were not associated with birth weight or childhood glucose concentrations, which both were previously found to be associated with maternal early-pregnancy glucose concentrations in our data (*p* values > 0.1) [9, 13].

### Look-up of maternal glucose metabolism and adult type 2 diabetes-associated CpGs

In a look-up in our results of CpGs and DMRs identified in previous studies to be associated with maternal glucose metabolism or with adult type 2 diabetes, we found that one CpG, cg1680945 at *MDN1*, known to be related to adult type 2 diabetes was also significantly associated with maternal early-pregnancy insulin concentrations (effect estimate = − 3.3 × 10^−3^ (SE 1.1 × 10^−3^), *p* value = 2.2 × 10^−3^) [27]. The look-up of other previously described CpGs and DMRs in the maternal early-pregnancy glucose and insulin EWAS results showed no associations (Additional file 8: Table S9; Additional file 9: Table S10; Additional File 10: Table S11; Additional file 11: Table S12) [1, 14, 17, 18, 22, 23, 25, 27–42]. We found enrichment for findings from one previous study on adult type 2 diabetes among the 24,935 nominally significant CpGs from the maternal early-pregnancy glucose EWAS results (Fisher combined probability *p* value = 0.04) [40]. No evidence for enrichment of the CpGs from other previous studies among the 24,935 nominally significant CpGs from the maternal early-pregnancy glucose cord blood analysis, nor among the 19,418 nominally significant CpGs from the maternal early-pregnancy insulin cord blood analysis was found (lowest Fisher combined probability *p* value = 0.15 in maternal early-pregnancy glucose EWAS results and *p* value = 0.12 in insulin EWAS results) [1, 14, 17, 18, 22, 23, 25, 27–42].

## Discussion

In this population-based EWAS we did not observe associations of maternal early-pregnancy glucose and insulin concentrations across the full spectrum with offspring cord blood DNA methylation in the full group. However, after stratification on maternal BMI, maternal early-pregnancy glucose concentrations were associated with DNA methylation at one CpG each among normal weight and among overweight or obese women. Associations of DNA methylation at these CpG sites did not persist in 10-year-old children. Also, we did not find associations with offspring health outcomes. Maternal early-pregnancy insulin was associated with one CpG known from a previous adult type 2 diabetes-associated study. Also, we found enrichment of CpGs identified in a previous EWAS on adult type 2 diabetes among our maternal early-pregnancy glucose EWAS results. Overall, our results constitute a first step toward a better understanding of a potential role of DNA methylation underlying the associations of maternal glycemic traits in early pregnancy with offspring health outcomes.

### Interpretation of main findings

Gestational diabetes or impaired glucose tolerance diagnosed in the second half of pregnancy increases the risks of adverse birth outcomes, of obesity and diabetes in the offspring [5, 6, 8]. It has been suggested that women who develop gestational diabetes or hyperglycemia later in pregnancy already have suboptimal glucose metabolism before or in early pregnancy [43, 44]. Maternal glycemic measures in early pregnancy have been described to be associated with altered fetal growth and glucose metabolism in childhood, but not with child adiposity, lipid levels, and blood pressure after adjustment for maternal pre-pregnancy BMI [9, 12, 13]. Thus, early pregnancy may already be a critical period for the effects of maternal glucose concentrations on offspring birth outcomes and glycemic health in childhood. The associations of maternal glucose metabolism with offspring outcomes may be explained by differential DNA methylation. Therefore, we hypothesized that maternal early-pregnancy glucose and insulin concentrations are associated with offspring DNA methylation levels at birth and that these associations may be different for normal weight and overweight women.

Results from a recent meta-analysis from seven pregnancy cohorts among 3677 mother-newborn pairs showed that gestational diabetes was not associated with differential methylation at a single CpG level, but it was associated with lower cord blood methylation levels within two specific regions [1]. In the current population-based EWASs, we did not find any associations in the full group, but maternal early-pregnancy glucose concentrations were associated with DNA methylation at cg03617420 (*XKR6*) among normal weight women, and at cg12081946 (*IL17D*) among overweight or obese women. The effect estimates of both CpGs were in opposite directions for normal weight and overweight or obese women, which could imply a modifying effect of maternal BMI, as we hypothesized.

*XKR6*, XK-related 6 gene, is located on chromosome 8 and is classified as a member of the Kell blood group complex subunit-related family. Genetic variants in this gene have previously been associated with type 2 diabetes, lipid concentrations, systolic blood pressure, and kidney function, among others [45–48]. *XKR6* is expressed in many tissues, most strongly in testis and lymphocytes, but also in the cerebellum and pancreas, among others. *IL17D*, interleukin 17D, is part of the cytokine family and located on chromosome 13 and has been previously associated with autoimmune and inflammatory diseases [49]. Autoimmune processes are part of the pathogenesis of type 1 diabetes mellitus [50]. Genetic variants close to *IL17D* have been associated with PR segment on electrocardiogram [51]. *IL17D* is most strongly expressed in the brain and skeletal muscle. Based on Roadmap Epigenomics Data Complete Collection extracted from the UCSC Genome Browser, both cg03617420 and cg12081946 coincide with DNAseI hypersensitivity clusters and transcription factor binding regions, indicating a location in potential regulatory elements.

DNA methylation levels at these CpGs have not been previously described in relation to maternal early-pregnancy glucose concentrations, and our results need replication in larger groups. We found that DNA methylation at one CpG, cg1680945 (*MDN1*), which was associated with adult type 2 diabetes in a previous study, was also associated with maternal early-pregnancy insulin concentrations [27]. We also found enrichment for CpGs identified in a previous EWAS on adult type 2 diabetes among the nominally significant CpGs from the maternal early-pregnancy glucose EWAS [40]. Maternal early-pregnancy glucose and insulin concentrations were not associated with any of the other previously reported maternal gestational diabetes, hyperglycemia or hyperinsulinemia, or adult type 2 diabetes-associated CpGs or differently methylated regions [1, 14, 17, 18, 22, 23, 25, 28–39, 41, 42].

The lack of identified associations in our total study group may have multiple reasons. Our study population is relatively healthy with on average lean women, with limited variability in maternal early-pregnancy glucose and insulin concentrations and with a low percentage of women who developed gestational diabetes. Associations of maternal early-pregnancy glucose and insulin concentrations with DNA methylation may be more apparent when using fasting glucose and insulin concentrations or among higher risk populations, as observed in studies in women with gestational diabetes [22, 31, 34]. Besides this, the moderate sample size of this study also limits the power to detect smaller differences. Another possibility is that associations of maternal early-pregnancy glucose and insulin concentrations with DNA methylation in offspring are more apparent in other tissues than cord blood, such as the placental tissue, body fat, skeletal muscle, liver, or pancreas. As shown in the analysis stratified on BMI, for some CpGs, associations may not be apparent in the full group as the directions of effect may be opposite between certain subgroups in the study population.

Maternal glucose concentrations were not associated with DNA methylation in child peripheral blood at age 10 years at the two CpGs identified in cord blood. This could imply a temporary effect of maternal glucose metabolism on offspring DNA methylation or it may be due to the relatively small sample size at age 10 years. Further studies should repeat these analyses among different age-groups in children to replicate our findings at birth and explore persistence of differential DNA methylation at the two identified CpGs. Also, the two identified CpGs were not associated with birth weight or childhood glucose concentrations in exploratory analyses. This may be due to the relatively small sample size or it may indicate that DNA methylation at these sites does not represent a biological pathway linking maternal glucose levels to birth weight or childhood glucose concentrations. Further studies, including studies among high-risk populations, are needed to examine the exact pathways involved in the associations of maternal glycemic measures during pregnancy with adverse birth outcomes and with offspring health outcomes, such as diabetes at later ages, in more detail.

This study suggests that maternal glycemic traits are associated with DNA methylation and that these associations may differ between mothers with overweight/obesity and those without. The role of the identified differential DNA methylation in pathways to offspring health needs further study. This is a first step toward discovering the underlying biological pathways and, if confirmed, it emphasizes the first trimester being a potentially important window of pregnancy for intervention studies to improve child health outcomes. Further, larger studies, with maternal early-pregnancy fasting blood samples, are needed to replicate our results.

### Methodological considerations

Major strengths of this study are the population-based prospective design and the fact that we have information on maternal plasma glucose and insulin concentrations in early pregnancy in combination with cord blood DNA methylation. In addition to single-CpG analyses, differential methylated regions were also evaluated. We were able to adjust for a large number of potential confounders and for estimated cell-type proportions. The relatively small number of mothers with gestational diabetes (1.3% versus 2–5% in the general Dutch population [52]) may be due to the fact that information on gestational diabetes was taken from medical records and there was no structural testing of all pregnant women. However, the low number of mothers with gestational diabetes and obesity included in the sample may also indicate a selection toward a healthy, non-diabetic and lean population that might influence the generalizability of our findings and may have limited our statistical power to detect significant associations. Glucose and insulin concentrations were measured once during early pregnancy. Future studies are needed to measure maternal glucose and insulin concentrations at multiple time points during pregnancy to observe whether normal glucose and insulin concentrations in early pregnancy will worsen or maintain normal during pregnancy and whether patterns of glucose and insulin concentrations during pregnancy may be more informative than single measurements. The blood samples in the study are non-fasting. They were collected after a fasting time of at least 30 min. Since glucose and insulin concentrations vary during the day and are sensitive to carbohydrate intake, this may have led to non-differential misclassification of glucose and insulin concentrations. However, it has been suggested that maternal non-fasting glucose concentrations may better reflect the normal physiological state in pregnancy [10, 53]. Ideally, data on oral glucose tolerance tests would have been included, but these are not available in the Generation R Study. Blood samples were collected and processed in a standardized way, but time from sampling to freezing could be up to four hours. This may have affected the measured glucose concentrations. DNA methylation was measured in blood, which may differ from methylation patterns in other tissues. The study participants are of European ancestry and therefore, the findings might not be generalizable to other populations.

## Conclusions

Maternal early-pregnancy blood glucose and insulin concentrations were not associated with differential DNA methylation at birth in the full group. However, maternal early-pregnancy glucose concentrations were associated with DNA methylation at one CpG in *XKR6* among normal weight women and at another CpG in *IL17D* among overweight or obese women. Their role in mechanisms underlying offspring health outcomes needs further study. These results await confirmation by future studies in larger samples with early-pregnancy information on maternal fasting glucose metabolism and exploring potential tissue-specific methylation effects in the offspring.

## Methods

### Study design

**Fig. 2:**
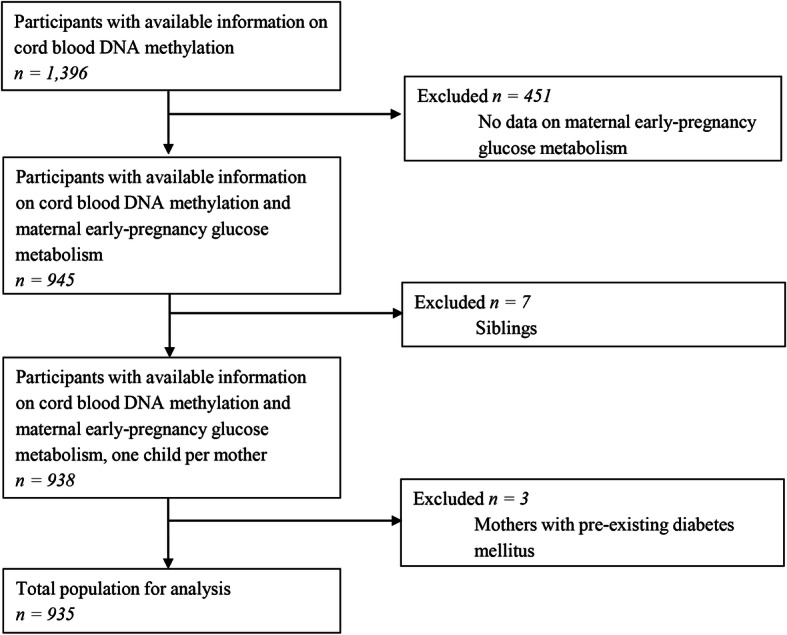
Study participants flowchart

This study was embedded in the Generation R Study, a population-based prospective cohort from early fetal life onwards, based in Rotterdam, the Netherlands [54]. The study has been approved by the Medical Ethical Committee of the Erasmus MC, University Medical Center Rotterdam (MEC 198.782/2001/31). Written informed consent was obtained for all participants [54]. In total, 8879 women were enrolled during pregnancy (response rate at baseline: 61%), of whom, 6186 had measurements of glucose and insulin concentrations available. DNA methylation was measured in cord blood of a randomly selected European-ancestry subset of *n* = 1396 mothers. Out of these mothers, *n* = 945 had measurements on early-pregnancy glucose metabolism available. We excluded women with pre-existing diabetes (*n* = 3); twin pregnancies and in case of multiple (non-twin) children per mother, we excluded one of each sibling pair, based on data completeness or, if equal, randomly (*n* = 7). The population for analysis of this study comprised 935 mother-newborn pairs (Fig. 2).

### Maternal glucose and insulin concentrations assessment

Blood samples were collected once in early pregnancy at 13.1 median weeks’ gestation (95% range 9.4–17.4), as described previously. After at least 30 min of fasting, venous blood samples were collected from pregnant women by research nurses and temporally stored at room temperature. Samples were minimally 30 min fasting. As we did not have information on the exact fasting duration, we consider all samples random. The time-interval of 30 min was chosen because of the design of the study, in which it was not possible to obtain fasting samples from all pregnant women. At least every 3 h, blood samples were transported to a dedicated laboratory facility of the regional laboratory in Rotterdam, the Netherlands (Star-MDC), for further processing and storage [55]. Glucose (mmol/l) was measured with the c702 module on a Cobas 8000 analyzer. Insulin (pmol/l) was measured with electrochemiluminescence immunoassay on a Cobas e411 analyzer. Quality control samples demonstrated intra- and inter-assay CVs of 0.9% and 1.2% for glucose concentrations and of 1.3% and 2.5% for insulin concentrations, respectively.

Information on pre-existing diabetes was obtained from self-reported questionnaires and on gestational diabetes from medical records after delivery. Gestational diabetes was diagnosed by a community midwife or an obstetrician according to Dutch midwifery and obstetric guidelines at the time of inclusion into the study, using the following criteria: either a random glucose concentration > 11.0 mmol/l, a fasting glucose ≥ 7.0 mmol/l, or a fasting glucose between 6.1 and 6.9 mmol/l with a subsequent abnormal glucose tolerance test [21].

### DNA methylation

DNA was extracted from cord blood using the salting-out method. Five hundred nanograms of DNA per sample underwent bisulfite conversion using the EZ-96 DNA Methylation kit (Shallow) (Zymo Research Corporation, Irvine, CA, USA). Samples were plated randomly onto 96-well plates. Samples were processed with the Illumina Infinium HumanMethylation450 (450 k) BeadChip (Illumina Inc., San Diego, CA, USA). Quality control of analyzed samples was performed using standardized criteria. Quality control and normalization of the array data was performed according to the Control Probe Adjustment and reduction of global CORrelation (CPACOR) workflow using R [56, 57]. Probes that had a detection *p* value ≥ 1E−16 were set to missing per array. Next, the intensity values were quantile normalized for each of the six probe-type categories separately: type II red/green, type I methylated red/green, and type I unmethylated red/green. Beta values were calculated as proportion of methylated intensity value to the sum of (methylated and unmethylated intensities plus 100). Arrays with observed technical problems such as failed bisulfite conversion, hybridization or extension, and arrays with a sex mismatch were removed from subsequent analyses. Additionally, only arrays with a call rate > 95% per sample were processed further. Probes on the X and Y chromosomes were excluded from the analyses. Outlying methylation beta values were excluded using the following method: values < (25th percentile – 3*interquartile range (3IQR)) and values > (75th percentile +3IQR) were removed [58]. For each analysis, we excluded plates with fewer than 3 samples because of convergence issues. This did not lead to exclusions in the analyses of the full group, but led to exclusion of 4 and 14 participants, in the normal weight and overweight/obese stratum, respectively. For all CpGs and differentially methylated regions, the official gene name of the nearest gene was noted using Illumina’s annotation information and we enhanced the annotation provided by Illumina with the UCSC Genome Browser build hg19 using the CpG location [59, 60].

### Covariates

Information on maternal age, pre-pregnancy weight, educational level, and parity was obtained from questionnaires at enrolment [61]. Maternal smoking during pregnancy was assessed by questionnaires in pregnancy. We measured maternal height at enrolment without shoes and heavy clothing. Pre-pregnancy BMI was calculated (self-reported pre-pregnancy weight in kilograms divided by height measured at enrolment in meters, squared). Information on gestational age at birth, child sex, and birth weight was obtained from medical records. To adjust for batch effects, plate number was included as a covariate in the analyses. We estimated leukocyte subtypes using a cord blood-specific reference [62]. This method estimates the relative proportions of six white blood cell subtypes (CD4+ T lymphocytes, CD8+ T lymphocytes, natural killer cells, B lymphocytes, monocytes, and granulocytes) and nucleated red blood cells.

### Statistical analysis

First, non-response analysis was conducted among participants with singleton children and information available on cord blood DNA methylation, comparing participants with to those without data on maternal early-pregnancy glucose metabolism available, using Student’s *t* tests, Mann-Whitney tests, and chi-square tests. Second, we used robust linear regression models in an EWAS framework to assess the associations of maternal early-pregnancy glucose and insulin concentrations with single-CpG DNA methylation in cord blood [57]. Maternal early-pregnancy insulin had a skewed distribution and was natural log-transformed for the analyses. The analyses were performed in two models: first model—adjusted for gestational age at glucose/insulin measurement, maternal age at intake, educational level, parity, smoking, child sex, cell-type proportions, and batch; and a secondary (main) model additionally adjusted for pre-pregnancy BMI. Since maternal obesity enhances the effect of higher glucose and insulin concentrations on adverse offspring outcomes, the effect on DNA methylation could be modified by maternal BMI. Therefore, as a secondary analysis, we stratified women into two strata of normal weight and overweight or obese women and repeated the main linear regression models in these strata. Included covariates were based on previous studies and factors known to be strongly associated with DNA methylation [22, 63–65]. Multiple testing was accounted for using Bonferroni correction, with CpGs with a *p* value < 1.0 × 10^−7^ considered significant. Additionally, we planned a priori to also report results using FDR correction for multiple testing, using the method by Benjamini and Hochberg [66]. Third, we identified differentially methylated regions using the dmrff package (https://github.com/perishky/dmrff), which identifies differentially methylated regions by combining EWAS summary statistics from nearby CpGs [26]. Significant differentially methylated regions were defined as regions spanning a set of CpG sites with at most 500 bp between consecutive sites with nominal EWAS *p* values < 0.05 and effect estimates with the same direction. Fourth, we examined whether associations of any CpGs identified in cord blood persisted in peripheral blood of 10-year-old children, using the main model additionally adjusted for child age at measurement. Missing covariate data were multiple-imputed using the Markov chain Monte Carlo method. All analyses were performed using R version 3.4.3 [57]. All authors had access to the study data and reviewed and approved the final manuscript.

### Associations of identified CpGs with offspring health outcomes

Exploratory analyses were performed in the relevant strata of maternal BMI to examine associations of identified CpGs with offspring birth weight and childhood glucose concentrations measured at age 10 years. We chose these outcomes because we have previously found them to be associated with maternal early-pregnancy glucose concentrations [9, 13]. We ran linear regression models using gestational age and sex-adjusted birth weight standard deviation scores (SDS) and childhood glucose concentrations as outcomes. Birth weight SDS were calculated based on the Niklasson reference charts, using Growth Analyzer (version 3.5; Dutch Growth Research Foundation, Rotterdam, the Netherlands) [67]. Models were adjusted for maternal age, educational level, parity, and smoking, as well as for plate number and the seven cell types from the cord blood reference [62]. Childhood glucose models were additionally adjusted for child sex and age at glucose measurement.

### Look-up of previously identified CpGs

We performed a look-up in our maternal early-pregnancy glucose and insulin results of previously described maternal glucose metabolism-associated CpGs and DMRs and of previously described adult type 2 diabetes-associated CpGs in adults. The PubMed search terms are described in Additional file 12: Note 1. We took those studies into account that (1) included more than 50 participants in total, (2) measured DNA methylation in cord blood or peripheral blood, (3) were epigenome-wide studies or candidate-gene studies, and (4) reported *p* values for single CpGs or differential methylated regions. Significance was determined based on a Bonferroni corrected *p* value < 0.05/number of tested CpGs per reference study. We also evaluated enrichment of these CpGs among CpGs with a *p* value < 0.05 in our EWAS results using a hypergeometric test.

## Supplementary information

**Additional file 1: Table S1.** Comparison of mother and child characteristics between participants included and non-participants.

**Additional file 2: Figure S1a.** Epigenome-wide association study results of maternal early-pregnancy glucose concentrations and DNA methylation in cord blood. **Figure S1b.** Epigenome-wide association study results of maternal early-pregnancy insulin concentrations and DNA methylation in cord blood.

**Additional file 3: Table S2.** CpGs with p-values <1.0 x 10^-4^ from epigenome-wide association study of maternal early-pregnancy glucose concentrations and DNA methylation. **Table S3.** CpGs with p-values <1.0 x 10^-4^ from epigenome-wide association study of maternal early-pregnancy insulin concentrations and DNA methylation.

**Additional file 4: Table S4.** CpGs with p-values <1.0 x 10^-4^ from epigenome-wide association study of maternal early-pregnancy glucose concentrations and DNA methylation - model without pre-pregnancy BMI adjustment. **Table S5. **CpGs with p-values <1.0 x 10^-4^ from epigenome-wide association study of maternal early-pregnancy insulin concentrations and DNA methylation - model without pre-pregnancy BMI adjustment.

**Additional file 5: Figure S2a.** Epigenome-wide association study results of maternal early-pregnancy insulin concentrations and DNA methylation in cord blood in normal weight women. **Figure S2b.** Epigenome-wide association study results of maternal early-pregnancy insulin concentrations and DNA methylation in cord blood in overweight or obese women.

**Additional file 6: Table S6.** Differentially Methylated Regions with p-values <1.0 x 10^-4^ associated with maternal early-pregnancy glucose concentrations. **Table S7.** Differentially Methylated Regions with p-values <1.0 x 10^-4^ associated maternal early-pregnancy insulin concentrations.

**Additional file 7: Table S8.** Look-up of two CpGs identified in cord blood in corresponding maternal BMI strata in epigenome-wide association study results of maternal early-pregnancy glucose concentrations and DNA methylation in 10-year-old children.

**Additional file 8: Table S9.** Look-up results of maternal glucose metabolism associated CpGs in maternal early-pregnancy glucose EWAS results.

**Additional file 9: Table S10.** Look-up results of maternal glucose metabolism associated CpGs in maternal early-pregnancy insulin EWAS results.

**Additional file 10: Table S11.** Look-up results of adult type 2 diabetes associated CpGs in maternal early-pregnancy glucose EWAS results.

**Additional file 11: Table S12.** Look-up results of adult type 2 diabetes associated CpGs in maternal early-pregnancy insulin EWAS results.

**Additional file 12: Note 1.**

## Data Availability

The datasets used and/or analyzed during the current study are available from the corresponding author on reasonable request.

## Abbreviations

BMI: Body mass index
CpGs: Cytosine-phosphate-guanine sites
DMR: Differentially methylated region
EWAS: Epigenome-wide association study
FDR: False-discovery rate
