# Supplementary material for: Associations of maternal early-pregnancy blood glucose and insulin concentrations with DNA methylation in newborns
Source: Clin Epigenetics. 2020 Sep 7;12:134. doi: 10.1186/s13148-020-00924-3 (PMC7487846; doi:10.1186/s13148-020-00924-3)
Supplement: Supplementary file 1 — Additional file 1: Table S1. Comparison of mother and child characteristics between participants included and non-participants. [file 13148_2020_924_MOESM1_ESM.docx]

**Table S1** Comparison of mother and child characteristics between participants included and non-participants

| Characteristics | Participants, *n = 935* | Non-participants, *n = 451* | p-value |
| --- | --- | --- | --- |
| Maternal characteristics |  |  |  |
| Age, years | 31.7 ± 4.2 | 31.8 ± 4.2 | 0.70 |
| Pre-pregnancy body mass index, kg/m^2^ | 23.2 ± 3.8 | 23.2 ± 3.9 | 0.74 |
| Gestational age at intake, weeks | 13.1 (9.8 – 22.2) | 13.1 (10.3 – 21.7) | 0.43 |
| Parity, nulliparous | 563 (60.3) | 279 (62.0) | 0.54 |
| Education, higher education | 596 (64.9) | 294 (65.8) | 0.74 |
| Smoking during pregnancy, continued | 173 (20.8) | 68 (20.1) | 0.77 |
| Child characteristics |  |  |  |
| Male | 491 (52.5) | 211 (46.8) | 0.05 |
| Gestational age at birth, weeks | 40.3 (36.4 – 42.3) | 40.3 (37.0 – 42.5) | 0.89 |
| Birth weight, g | 3552 ± 514 | 3522 ± 503 | 0.29 |

Values are means ± SD, medians (95% range) or numbers of subjects (valid %). Differences were tested using Student’s t-tests and Mann-Whitney tests for normally and non-normally distributed variables, respectively, and χ2-test were used for dichotomous variables.
