# Supplementary material for: Associations of maternal early-pregnancy blood glucose and insulin concentrations with DNA methylation in newborns
Source: Clin Epigenetics. 2020 Sep 7;12:134. doi: 10.1186/s13148-020-00924-3 (PMC7487846; doi:10.1186/s13148-020-00924-3)
Supplement: Supplementary file 2 — Additional file 2: Figure S1a. Epigenome-wide association study results of maternal early-pregnancy glucose concentrations and DNA methylation in cord blood. Figure S1b. Epigenome-wide association study results of maternal early-pregnancy insulin concentrations and DNA methylation in cord blood. [file 13148_2020_924_MOESM2_ESM.docx]

**Figure S1a** Epigenome-wide association study results of maternal early-pregnancy glucose concentrations and DNA methylation in cord blood
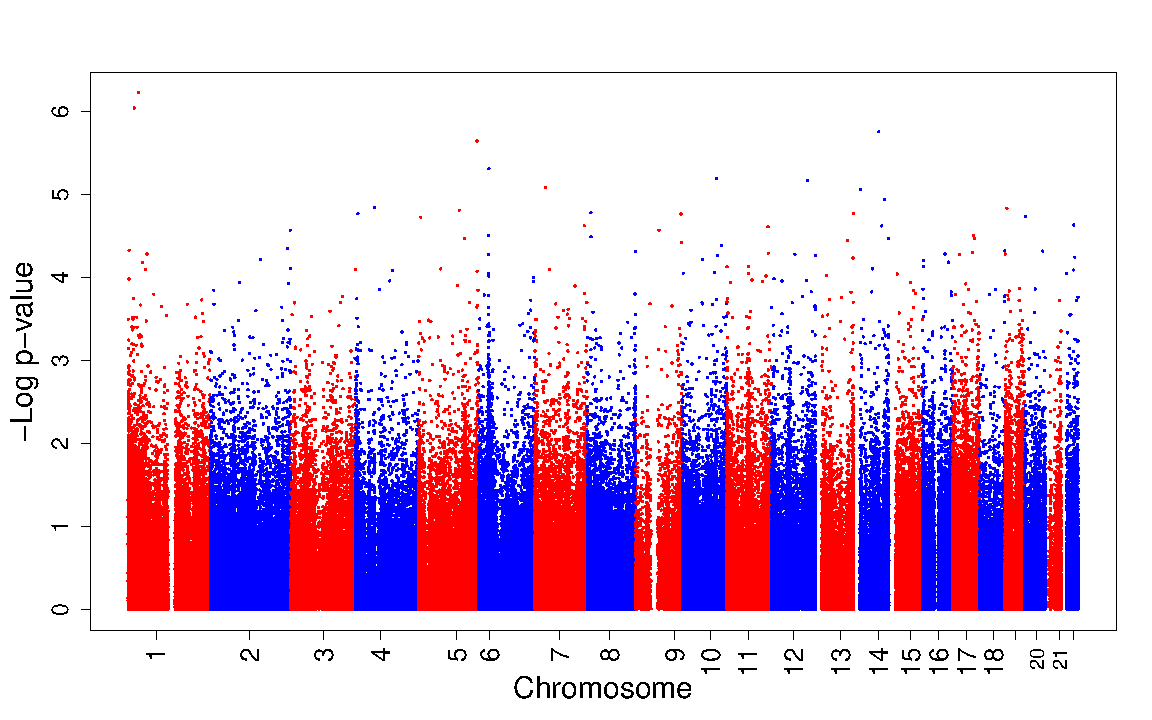


**Figure S1b** Epigenome-wide association study results of maternal early-pregnancy insulin concentrations and DNA methylation in cord blood


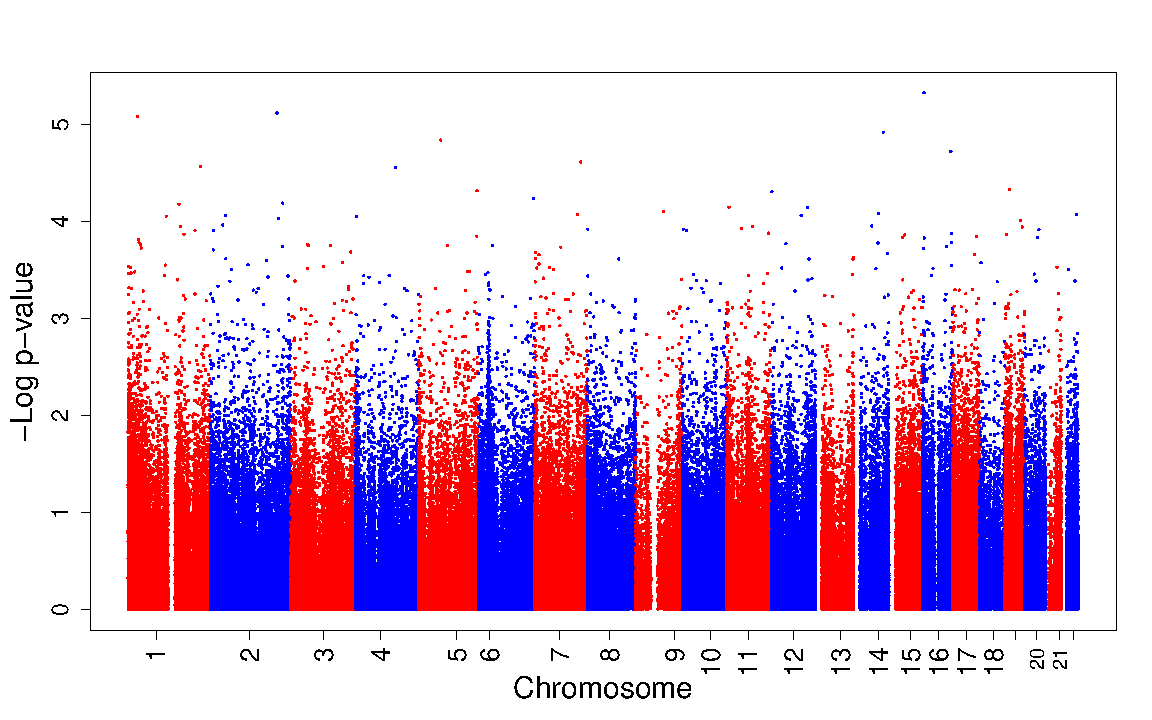


In 1a. the Manhattan plot shows the results of the epigenome-wide association study of maternal early-pregnancy glucose concentrations and DNA methylation in cord blood in 935 newborns. In 1b. the Manhattan plot shows the results of the epigenome-wide association study of maternal early-pregnancy insulin concentrations and DNA methylation in cord blood in 935 newborns IN both figures the x-axis represents the autosomal (1 – 22) chromosomes and the y-axis shows the –log_10_ (p-value). The models were adjusted for gestational age at assessment, maternal age at intake, educational level, parity, smoking, pre-pregnancy BMI, child sex, cell type proportions and batch.
