# Supplementary material for: Associations of maternal early-pregnancy blood glucose and insulin concentrations with DNA methylation in newborns
Source: Clin Epigenetics. 2020 Sep 7;12:134. doi: 10.1186/s13148-020-00924-3 (PMC7487846; doi:10.1186/s13148-020-00924-3)
Supplement: Supplementary file 4 — Additional file 4: Table S4. CpGs with p-values <1.0 x 10^-4^ from epigenome-wide association study of maternal early-pregnancy glucose concentrations and DNA methylation - model without pre-pregnancy BMI adjustment. Table S5. CpGs with p-values <1.0 x 10^-4^ from epigenome-wide association study of maternal early-pregnancy insulin concentrations and DNA methylation - model without pre-pregnancy BMI adjustment. [file 13148_2020_924_MOESM4_ESM.docx]

**Table S4** CpGs with p-values < 1.0 x 10^-4^ from epigenome-wide association study of maternal early-pregnancy glucose concentrations and DNA methylation - model without pre-pregnancy BMI adjustment

| CpG | Chr | Position | Gene | Effect | SE | P-value |
| --- | --- | --- | --- | --- | --- | --- |
| cg19225369 | 1 | 32255601 | *RP11-84A19.3* | -3.17 x 10^-3^ | 6.37 x 10^-4^ | 6.35 x 10^-7^ |
| cg19872936 | 14 | 76932055 | *ESRRB* | 6.54 x 10^-3^ | 1.32 x 10^-3^ | 7.73 x 10^-7^ |
| cg08164334 | 1 | 19229453 | *ALDH4A1* | -1.39 x 10^-3^ | 2.83 x 10^-4^ | 9.07 x 10^-7^ |
| cg09013267 | 5 | 176762943 | *LMAN2* | -3.46 x 10^-3^ | 7.26 x 10^-4^ | 1.89 x 10^-6^ |
| cg12647920 | 12 | 109144744 | *CORO1C* | -3.81 x 10^-3^ | 8.27 x 10^-4^ | 4.01 x 10^-6^ |
| cg22538475 | 6 | 32094102 | *ATF6B* | -1.99 x 10^-3^ | 4.44 x 10^-4^ | 7.51 x 10^-6^ |
| cg07981355 | 10 | 104837141 | *CNNM2* | -2.25 x 10^-3^ | 5.03 x 10^-4^ | 7.56 x 10^-6^ |
| cg08445039 | 7 | 32997659 | *FKBP9* | 5.71 x 10^-3^ | 1.28 x 10^-3^ | 8.09 x 10^-6^ |
| cg16534427 | 14 | 21550253 | *FLJ10357* | -3.22 x 10^-3^ | 7.32 x 10^-4^ | 1.10 x 10^-5^ |
| cg10313093 | 4 | 56261753 | *TMEM165* | 1.50 x 10^-3^ | 3.42 x 10^-4^ | 1.17 x 10^-5^ |
| cg13487983 | 9 | 137296319 | *RXRA* | 3.79 x 10^-3^ | 8.69 x 10^-4^ | 1.30 x 10^-5^ |
| cg16244889 | 14 | 94211818 | *PRIMA1* | 3.89 x 10^-3^ | 8.93 x 10^-4^ | 1.33 x 10^-5^ |
| cg05489065 | 5 | 6381180 | *MED10* | -2.37 x 10^-3^ | 5.48 x 10^-4^ | 1.49 x 10^-5^ |
| cg11570367 | 13 | 114896683 | *RASA3* | -2.59 x 10^-3^ | 5.99 x 10^-^**^4^** | 1.51 x 10^-5^ |
| cg12233487 | 20 | 2517085 | *TMC2* | 8.85 x 10^-3^ | 2.05 x 10^-3^ | 1.55 x 10^-5^ |
| cg11207954 | 19 | 6002692 | *RFX2* | -2.74 x 10^-3^ | 6.38 x 10^-4^ | 1.68 x 10^-5^ |
| cg27059389 | 8 | 10855370 | *XKR6* | -8.45 x 10^-3^ | 1.97 x 10^-3^ | 1.76 x 10^-5^ |
| cg00086871 | 4 | 6988644 | *TBC1D14* | 1.66 x 10^-3^ | 3.88 x 10^-4^ | 1.80 x 10^-5^ |
| cg23992152 | 5 | 122971768 | *KRT18P16* | -3.09 x 10^-3^ | 7.22 x 10^-4^ | 1.83 x 10^-5^ |
| cg03617420 | 8 | 10916666 | *XKR6* | 4.81 x 10^-3^ | 1.13 x 10^-3^ | 1.98 x 10^-5^ |
| cg04146259 | 14 | 85058259 | *RNU6-976P* | -2.23 x 10^-3^ | 5.25 x 10^-4^ | 2.19 x 10^-5^ |
| cg19360907 | 22 | 37977445 | *LGALS2* | -9.28 x 10^-3^ | 2.19 x 10^-3^ | 2.27 x 10^-5^ |
| cg04898026 | 7 | 149487677 | *SSPO* | 4.43 x 10^-3^ | 1.06 x 10^-3^ | 2.82 x 10^-5^ |
| cg12255061 | 17 | 63533152 | *AXIN2* | 2.77 x 10^-3^ | 6.62 x 10^-4^ | 2.92 x 10^-5^ |
| cg03885639 | 11 | 124481310 | *PANX3* | -3.07 x 10^-3^ | 7.36 x 10^-4^ | 3.08 x 10^-5^ |
| cg05157340 | 17 | 67577736 | *LINC01483* | 2.11 x 10^-3^ | 5.08 x 10^-4^ | 3.11 x 10^-5^ |
| cg24040299 | 14 | 105834764 | *PACS2* | -3.14 x 10^-3^ | 7.56 x 10^-4^ | 3.22 x 10^-5^ |
| cg21475834 | 13 | 96706104 | *UGGT2* | -6.39 x 10^-3^ | 1.54 x 10^-3^ | 3.27 x 10^-5^ |
| cg13007349 | 5 | 137911196 | *HSPA9* | -9.65 x 10^-4^ | 2.34 x 10^-4^ | 3.68 x 10^-5^ |
| cg26459312 | 9 | 70971731 | *LOC572558, PGM5* | 6.42 x 10^-3^ | 1.56 x 10^-3^ | 3.77 x 10^-5^ |
| cg17536532 | 9 | 138370739 | *KIAA0649* | 4.13 x 10^-3^ | 1.00 x 10^-3^ | 3.80 x 10^-5^ |
| cg16654801 | 10 | 119301763 | *EMX2, EMX2OS* | -1.75 x 10^-3^ | 4.27 x 10^-4^ | 4.07 x 10^-5^ |
| cg04979655 | 2 | 234358327 | *DGKD* | -1.22 x 10^-3^ | 2.98 x 10^-4^ | 4.32 x 10^-5^ |
| cg00065088 | 8 | 144943738 | *EPPK1* | -2.37 x 10^-3^ | 5.82 x 10^-4^ | 4.50 x 10^-5^ |
| cg10605681 | 6 | 30228153 | *HLA-L* | 3.48 x 10^-3^ | 8.54 x 10^-4^ | 4.52 x 10^-5^ |
| cg17772649 | 6 | 31691426 | *C6orf25* | 6.04 x 10^-3^ | 1.48 x 10^-3^ | 4.64 x 10^-5^ |
| cg11804932 | 10 | 106402507 | *SORCS3* | 1.91 x 10^-3^ | 4.69 x 10^-4^ | 4.76 x 10^-5^ |
| cg16881309 | 2 | 242202048 | *HDLBP* | -2.26 x 10^-3^ | 5.57 x 10^-4^ | 4.86 x 10^-5^ |
| cg03928182 | 12 | 71314662 | *PTPRR* | -3.37 x 10^-3^ | 8.30 x 10^-4^ | 4.87 x 10^-5^ |
| cg06705017 | 18 | 77552402 | *RP11-154H12.3* | 8.32 x 10^-3^ | 2.05 x 10^-3^ | 4.91 x 10^-5^ |
| cg18369939 | 1 | 3239916 | *PRDM16* | 5.02 x 10^-3^ | 1.24 x 10^-3^ | 4.99 x 10^-5^ |
| cg03384825 | 1 | 57889604 | *DAB1* | 1.09 x 10^-3^ | 2.69 x 10^-4^ | 5.14 x 10^-5^ |
| cg08720806 | 11 | 125142671 | *PKNOX2* | 5.39 x 10^-3^ | 1.33 x 10^-3^ | 5.17 x 10^-5^ |
| cg26168577 | 17 | 60766925 | *MRC2* | -1.38 x 10^-3^ | 3.41 x 10^-4^ | 5.18 x 10^-5^ |
| cg16623362 | 12 | 133161907 | *FBRSL1* | 5.67 x 10^-3^ | 1.40 x 10^-3^ | 5.21 x 10^-5^ |
| cg12230709 | 19 | 840873 | *PRTN3* | 5.15 x 10^-3^ | 1.27 x 10^-3^ | 5.23 x 10^-5^ |
| cg26876114 | 16 | 2511507 | *C16orf59* | -2.54 x 10^-3^ | 6.27 x 10^-4^ | 5.28 x 10^-5^ |
| cg19091902 | 17 | 21415527 | *RPL21P120* | 5.53 x 10^-3^ | 1.37 x 10^-3^ | 5.35 x 10^-5^ |
| cg24722198 | 13 | 113567732 | *MCF2L* | -4.22 x 10^-3^ | 1.05 x 10^-3^ | 5.55 x 10^-5^ |
| cg09899094 | 7 | 44122310 | *POLM* | -1.32 x 10^-3^ | 3.27 x 10^-4^ | 5.58 x 10^-5^ |
| cg13227473 | 10 | 62538143 | *CDK1,CDC2* | 1.93 x 10^-3^ | 4.83 x 10^-4^ | 6.16 x 10^-5^ |
| cg16586336 | 16 | 2748071 | *KCTD5* | -2.72 x 10^-3^ | 6.80 x 10^-4^ | 6.18 x 10^-5^ |
| cg24699418 | 1 | 44411974 | *IPO13* | 1.77 x 10^-3^ | 4.44 x 10^-4^ | 6.40 x 10^-5^ |
| cg06494642 | 16 | 67233861 | *ELMO3* | 3.83 x 10^-3^ | 9.58 x 10^-4^ | 6.46 x 10^-5^ |
| cg03279185 | 16 | 78082781 | *CLEC3A, RP11-281J9.2* | -2.47 x 10^-3^ | 6.19 x 10^-4^ | 6.47 x 10^-5^ |
| cg20123649 | 11 | 66629353 | *PC* | -3.18 x 10^-3^ | 8.00 x 10^-4^ | 7.04 x 10^-5^ |
| cg24512778 | 2 | 152684544 | *ARL5A* | 1.60 x 10^-3^ | 4.03 x 10^-4^ | 7.06 x 10^-5^ |
| cg10266121 | 10 | 97020570 | *PDLIM1* | -2.45 x 10^-3^ | 6.16 x 10^-4^ | 7.06 x 10^-5^ |
| cg04882349 | 1 | 53098299 | *FAM159A* | 3.50 x 10^-3^ | 8.82 x 10^-4^ | 7.19 x 10^-5^ |
| cg14906510 | 12 | 7781169 | *APOBEC1* | -6.24 x 10^-3^ | 1.57 x 10^-3^ | 7.33 x 10^-5^ |
| cg04444873 | 20 | 54967407 | *AURKA, CSTF1* | 7.32 x 10^-4^ | 1.85 x 10^-4^ | 7.44 x 10^-5^ |
| cg21627187 | 3 | 196751186 | *MFI2* | 4.56 x 10^-3^ | 1.15 x 10^-3^ | 7.69 x 10^-5^ |
| cg23950714 | 5 | 176935364 | *DOK3* | 7.20 x 10^-3^ | 1.82 x 10^-3^ | 7.70 x 10^-5^ |
| cg08761208 | 15 | 65693289 | *IGDCC4* | 6.34 x 10^-3^ | 1.60 x 10^-3^ | 7.90 x 10^-5^ |
| cg19458529 | 5 | 66347164 | *MAST4* | -3.69 x 10^-3^ | 9.37 x 10^-4^ | 8.04 x 10^-5^ |
| cg00735843 | 14 | 57735967 | *MUDENG, EXOC5* | 2.44 x 10^-3^ | 6.21 x 10^-4^ | 8.48 x 10^-5^ |
| cg04350311 | 22 | 37821982 | *ELFN2* | 6.00 x 10^-3^ | 1.53 x 10^-3^ | 8.49 x 10^-5^ |
| cg15055810 | 12 | 120650476 | *PXN* | -2.35 x 10^-3^ | 5.99 x 10^-3^ | 8.60 x 10^-5^ |
| cg08711796 | 22 | 16287910 | *POTEH* | -3.63 x 10^-3^ | 9.24 x 10^-4^ | 8.71 x 10^-5^ |
| cg04222344 | 2 | 242445904 | *STK25* | -2.01 x 10^-3^ | 5.12 x 10^-4^ | 8.86 x 10^-5^ |
| cg12908429 | 15 | 25306290 | *SNORD116-5, SNORD116-7* | -4.37 x 10^-3^ | 1.12 x 10^-3^ | 8.95 x 10^-5^ |
| cg16014770 | 12 | 31882421 | *AMN1* | -2.55 x 10^-3^ | 6.51 x 10^-4^ | 9.20 x 10^-5^ |
| cg13464738 | 6 | 30950779 | *MUC21* | 3.94 x 10^-3^ | 1.01 x 10^-3^ | 9.31 x 10^-5^ |
| cg03743753 | 16 | 86575852 | *MTHFSD* | -9.83 x 10^-4^ | 2.52 x 10^-4^ | 9.47 x 10^-5^ |
| cg14698932 | 13 | 34088638 | *RP11-141M1.3* | -2.62 x 10^-3^ | 6.72 x 10^-4^ | 9.53 x 10^-5^ |
| cg19547141 | 11 | 118313315 | *MLL* | -2.91 x 10^-3^ | 7.47 x 10^-4^ | 9.63 x 10^-5^ |

Effect estimates represent the difference in DNA methylation per 1 mmol/l increase in maternal early-pregnancy glucose concentrations. The model was adjusted for gestational age at assessment, maternal age at intake, educational level, parity, smoking, child sex, cell type proportions and batch. Chr, chromosome; SE, standard error.

**Table S5** CpGs with p-values < 1.0 x 10^-4^ from epigenome-wide association study of maternal early-pregnancy insulin concentrations and DNA methylation - model without pre-pregnancy BMI adjustment

| CpG | Chr | Position | Gene | Effect | SE | P-value |
| --- | --- | --- | --- | --- | --- | --- |
| cg08445323 | 16 | 4015030 | *ADCY9* | -2.46 x 10^-3^ | 5.31 x 10^-4^ | 3.56 x 10^-6^ |
| cg15166089 | 1 | 27710030 | *CD164L2* | 3.47 x 10^-3^ | 7.71 x 10^-4^ | 6.75 x 10^-6^ |
| cg02738983 | 2 | 202315807 | *TRAK2, STRADB* | -1.54 x 10^-3^ | 3.46 x 10^-4^ | 8.35 x 10^-6^ |
| cg24714561 | 14 | 90001131 | *FOXN3* | -2.02 x 10^-3^ | 4.64 x 10^-4^ | 1.34 x 10^-5^ |
| cg19458529 | 5 | 66347164 | *MAST4* | -3.72 x 10^-3^ | 8.62 x 10^-4^ | 1.60 x 10^-5^ |
| cg00834923 | 16 | 85393998 | *RP11-680G10.1* | 4.19 x 10^-3^ | 9.79 x 10^-4^ | 1.87 x 10^-5^ |
| cg17198017 | 7 | 139305268 | *HIPK2* | -1.96 x 10^-3^ | 4.63 x 10^-4^ | 2.38 x 10^-5^ |
| cg06807791 | 1 | 220232018 | *BPNT1* | 3.96 x 10^-3^ | 9.41 x 10^-4^ | 2.52 x 10^-5^ |
| cg04279596 | 4 | 120133691 | *USP53* | -8.75 x 10^-4^ | 2.12 x 10^-4^ | 3.66 x 10^-5^ |
| cg15939937 | 19 | 14676202 | *TECR* | 2.98 x 10^-3^ | 7.30 x 10^-4^ | 4.38 x 10^-5^ |
| cg05691934 | 12 | 1755162 | *WNT5B* | 2.79 x 10^-3^ | 6.87 x 10^-4^ | 4.88 x 10^-5^ |
| cg17797360 | 6 | 167792741 | *TCP10* | -4.65 x 10^-3^ | 1.14 x 10^-3^ | 4.95 x 10^-5^ |
| cg16114706 | 22 | 46509464 | *LOC400931, MIRLET7B* | -2.88 x 10^-3^ | 7.21 x 10^-4^ | 6.34 x 10^-5^ |
| cg26678978 | 11 | 6280531 | *CCKBR* | -1.81 x 10^-3^ | 4.53 x 10^-4^ | 6.59 x 10^-5^ |
| cg08621957 | 2 | 219181531 | *PNKD* | -4.12 x 10^-3^ | 1.03 x 10^-3^ | 6.70 x 10^-5^ |
| cg13096330 | 1 | 154546517 | *CHRNB2* | -4.31 x 10^-3^ | 1.08 x 10^-3^ | 6.77 x 10^-5^ |
| cg19027636 | 5 | 176558658 | *NSD1* | 2.40 x 10^-3^ | 6.08 x 10^-4^ | 7.89 x 10^-5^ |
| cg04237618 | 1 | 115829610 | *NGF* | -2.96 x 10^-3^ | 7.52 x 10^-4^ | 8.18 x 10^-5^ |
| cg19690404 | 7 | 129933646 | *CPA4* | -4.86 x 10^-3^ | 1.23 x 10^-3^ | 8.22 x 10^-5^ |
| cg22714942 | 12 | 109911508 | *KCTD10* | 1.84 x 10^-3^ | 4.67 x 10^-4^ | 8.28 x 10^-5^ |
| cg02315315 | 2 | 47055138 | *LOC100134259* | -2.73 x 10^-3^ | 6.93 x 10^-4^ | 8.31 x 10^-5^ |
| cg21238002 | 9 | 83496248 | *RP11-289F5.1* | 3.41 x 10^-3^ | 8.67 x 10^-4^ | 8.40 x 10^-5^ |
| cg14416248 | 4 | 3170095 | *HTT* | -3.25 x 10^-3^ | 8.32 x 10^-4^ | 9.24 x 10^-5^ |
| cg16960402 | 7 | 2032075 | *MAD1L1* | 3.05 x 10^-3^ | 7.83 x 10^-4^ | 9.64 x 10^-5^ |
| cg15488122 | 19 | 46105590 | *GPR4* | 2.67 x 10^-3^ | 6.85 x 10^-4^ | 9.73 x 10^-5^ |

Effect estimates represent the difference in DNA methylation per 1 pmol/l increase in maternal early-pregnancy natural log-transformed insulin concentrations. The model was adjusted for gestational age at assessment, maternal age at intake, educational level, parity, smoking, child sex, cell type proportions and batch. Chr, chromosome; SE, standard error.
