# Supplementary material for: Associations of maternal early-pregnancy blood glucose and insulin concentrations with DNA methylation in newborns
Source: Clin Epigenetics. 2020 Sep 7;12:134. doi: 10.1186/s13148-020-00924-3 (PMC7487846; doi:10.1186/s13148-020-00924-3)
Supplement: Supplementary file 6 — Additional file 6: Table S6. Differentially Methylated Regions with p-values <1.0 x 10^-4^ associated with maternal early-pregnancy glucose concentrations. Table S7. Differentially Methylated Regions with p-values <1.0 x 10^-4^ associated maternal early-pregnancy insulin concentrations. [file 13148_2020_924_MOESM6_ESM.docx]

**Table S6** Differentially Methylated Regions with p-values < 1.0 x 10^-4^ associated with maternal early-pregnancy glucose concentrations

| Chr | Start | End | N probes | Gene | Effect | SE | P-value |
| --- | --- | --- | --- | --- | --- | --- | --- |
| 4 | 6988644 | 6988698 | 2 | *TBC1D14* | 1.59 x 10^-3^ | 3.31 x 10^-4^ | 1.65 x 10^-6^ |
| 10 | 104836841 | 104837141 | 3 | *CNNM2* | -1.89 x 10^-3^ | 4.00 x 10^-4^ | 2.39 x 10^-6^ |
| 7 | 32997590 | 32997659 | 2 | *FKBP9, AVL9* | 3.05 x 10^-3^ | 6.86 x 10^-4^ | 8.81 x 10^-6^ |
| 19 | 46999403 | 46999444 | 2 | *PPP5D1* | 2.93 x 10^-3^ | 6.72 x 10^-4^ | 1.36 x 10^-5^ |
| 3 | 52188418 | 52188725 | 4 | *POC1A* | -7.32 x 10^-4^ | 1.68 x 10^-4^ | 1.36 x 10^-5^ |
| 8 | 144242106 | 144242448 | 5 | *LY6H* | 1.59 x 10^-3^ | 3.73 x 10^-4^ | 2.06 x 10^-5^ |
| 7 | 149487677 | 149487749 | 2 | *SSPO* | 4.23 x 10^-3^ | 1.00 x 10^-3^ | 2.38 x 10^-5^ |
| 16 | 67562424 | 67562517 | 2 | *FAM65A* | 2.70 x 10^-3^ | 6.39 x 10^-4^ | 2.38 x 10^-5^ |
| 13 | 113719136 | 113719440 | 3 | *MCF2L* | -9.15 x 10^-4^ | 2.19 x 10^-4^ | 3.06 x 10^-5^ |
| 2 | 74882001 | 74882132 | 2 | *SEMA4F* | 3.38 x 10^-3^ | 8.10 x 10^-4^ | 3.08 x 10^-5^ |
| 18 | 77552402 | 77552568 | 2 | *RP11-154H12.2, RP11-154H12.3* | 8.20 x 10^-3^ | 1.97 x 10^-3^ | 3.12 x 10^-5^ |
| 7 | 1923537 | 1923613 | 2 | *MAD1L1* | -2.18 x 10^-3^ | 5.25 x 10^-4^ | 3.24 x 10^-5^ |
| 19 | 22715410 | 22715697 | 3 | *LINC01233* | 3.39 x 10^-3^ | 8.20 x 10^-4^ | 3.46 x 10^-5^ |
| 3 | 73674339 | 73674656 | 5 | *PDZRN3-AS1* | 1.92 x 10^-3^ | 4.66 x 10^-4^ | 3.57 x 10^-5^ |
| 17 | 37123669 | 37123711 | 3 | *FBXO47* | 6.07 x 10^-3^ | 1.48 x 10^-3^ | 3.97 x 10^-5^ |
| 13 | 114066988 | 114067394 | 3 | *ADRPHL1* | 4.97 x 10^-4^ | 1.21 x 10^-4^ | 4.49 x 10^-5^ |
| 12 | 7781169 | 7781288 | 2 | *NIFKP3, RP11-444J21.2* | -6.59 x 10^-3^ | 1.61 x 10^-3^ | 5.02 x 10^-5^ |
| 6 | 30228083 | 30228153 | 3 | *HCG17, HLA-L* | 2.96 x 10^-3^ | 7.29 x 10^-4^ | 5.21 x 10^-5^ |
| 6 | 30881645 | 30881728 | 4 | *GTF2H4* | 5.61 x 10^-3^ | 1.39 x 10^-3^ | 6.00 x 10^-5^ |
| 7 | 100797533 | 100797595 | 3 | *AP1S1* | 7.73 x 10^-4^ | 1.93 x 10^-4^ | 6.36 x 10^-5^ |
| 12 | 123237179 | 123237461 | 3 | *DENR* | -5.11 x 10^-4^ | 1.28 x 10^-4^ | 6.45 x 10^-5^ |
| 5 | 102091118 | 102091191 | 2 | *PAM* | 1.71 x 10^-3^ | 4.27 x 10^-4^ | 6.46 x 10^-5^ |
| 14 | 94789681 | 94789853 | 3 | *SERPINA6* | -2.80 x 10^-3^ | 7.12 x 10^-4^ | 8.46 x 10^-5^ |
| 18 | 34834605 | 34834690 | 2 | *CELF4* | 4.82 x 10^-3^ | 1.23 x 10^-3^ | 8.48 x 10^-5^ |
| 4 | 77996437 | 77996840 | 3 | *CCNI* | 5.74 x 10^-4^ | 1.46 x 10^-4^ | 8.90 x 10^-5^ |
| 7 | 1120003 | 1120141 | 2 | *C7orf50* | 1.09 x 10^-3^ | 2.79 x 10^-4^ | 9.59 x 10^-5^ |
| 7 | 150780510 | 150780872 | 5 | *TMUB1* | 4.46 x 10^-4^ | 1.15 x 10^-4^ | 9.90 x 10^-5^ |

Results present identified differentially methylated regions from association analyses of maternal early-pregnancy glucose concentrations and represent the difference in DNA methylation per 1 mmol/l change in maternal early-pregnancy glucose concentrations. The model was adjusted for gestational age at assessment, maternal age at intake, educational level, parity, smoking, pre-pregnancy BMI, child sex, cell type proportions and batch. Chr, chromosome; SE, standard error.

**Table S7** Differentially Methylated Regions with p-values < 1.0 x 10^-4^ associated maternal early-pregnancy insulin concentrations

| Chr | Start | End | N of probes | Gene | Effect | SE | P-value |
| --- | --- | --- | --- | --- | --- | --- | --- |
| 7 | 56160687 | 56160717 | 2 | *PHKG1* | 4.26 x 10^-3^ | 9.63 x 10^-4^ | 9.81 x 10^-6^ |
| 3 | 175338261 | 175338357 | 2 | *NAALADL2* | -2.16 x 10^-3^ | 4.88 x 10^-4^ | 9.88 x 10^-6^ |
| 7 | 1740190 | 1740507 | 3 | *ELFN1* | 1.98 x 10^-3^ | 4.64 x 10^-4^ | 2.04 x 10^-5^ |
| 3 | 194392930 | 194393231 | 5 | *LSG1* | -4.08 x 10^-4^ | 9.59 x 10^-5^ | 2.13 x 10^-5^ |
| 9 | 17906338 | 17906561 | 2 | *SH3GL2* | -1.37 x 10^-3^ | 3.25 x 10^-4^ | 2.56 x 10^-5^ |
| 2 | 107199044 | 107199102 | 2 | *AC108868.6* | -1.77 x 10^-3^ | 4.41 x 10^-4^ | 5.98 x 10^-5^ |
| 7 | 157346224 | 157346614 | 2 | *PTPRN2* | 1.83 x 10^-3^ | 4.70 x 10^-3^ | 9.45 x 10^-5^ |
| 6 | 28870818 | 28871376 | 3 | *TRIM27* | 1.37 x 10^-3^ | 3.52 x 10^-4^ | 9.65 x 10^-5^ |
| 1 | 46152339 | 46152368 | 2 | *GPBP1L1* | -4.67 x 10^-4^ | 1.20 x 10^-4^ | 9.81 x 10^-5^ |

Results present identified differentially methylated regions from association analyses of maternal early-pregnancy insulin concentrations and represent the difference in DNA methylation per 1 pmol/l change in maternal early-pregnancy natural log-transformed insulin concentration. The model was adjusted for gestational age at glucose/insulin measurement, maternal age at intake, educational level, parity, smoking, pre-pregnancy BMI, child sex, cell type proportions and batch. Chr, chromosome; SE, standard error.
