# Supplementary material for: Associations of maternal early-pregnancy blood glucose and insulin concentrations with DNA methylation in newborns
Source: Clin Epigenetics. 2020 Sep 7;12:134. doi: 10.1186/s13148-020-00924-3 (PMC7487846; doi:10.1186/s13148-020-00924-3)
Supplement: Supplementary file 7 — Additional file 7: Table S8. Look-up of two CpGs identified in cord blood in corresponding maternal BMI strata in epigenome-wide association study results of maternal early-pregnancy glucose concentrations and DNA methylation in 10-year-old children. [file 13148_2020_924_MOESM7_ESM.docx]

**Table S8.** Look-up of two CpGs identified in cord blood in corresponding maternal BMI strata in epigenome-wide association study results of maternal early-pregnancy glucose concentrations and DNA methylation in 10-year-old children

| CpG | Chr | Position | Gene | Effect | SE | P-value |
| --- | --- | --- | --- | --- | --- | --- |
| cg03617420 | 8 | 10916666 | *XKR6* | -4.00 x 10^-3^ | 2.03 x 10^-3^ | 0.049 |
| cg12081946 | 13 | 21296465 | *IL17D* | -2.56 x 10^-3^ | 8.99 x 10^-3^ | 0.776 |

Effect estimates represent the difference in DNA methylation per 1 mmol/l increase in maternal early-pregnancy glucose concentrations. The model was adjusted for gestational age at assessment, maternal age at intake, educational level, parity, smoking, pre-pregnancy BMI, child sex, child age at assessement, cell type proportions and batch. Chr, chromosome; SE, standard error.
