# Supplementary material for: Associations of maternal early-pregnancy blood glucose and insulin concentrations with DNA methylation in newborns
Source: Clin Epigenetics. 2020 Sep 7;12:134. doi: 10.1186/s13148-020-00924-3 (PMC7487846; doi:10.1186/s13148-020-00924-3)
Supplement: Supplementary file 12 — Additional file 12: Note 1. [file 13148_2020_924_MOESM12_ESM.docx]

**Supplementary Material Note 1**

Pubmed publisher maternal glucose and insulin or gestational diabetes associated CpGs:

(("Epigenomics"[mh] OR "Epigenesis, Genetic"[mh] OR "DNA methylation"[mh] OR "CpG Islands"[mh] OR "methylat*"[tiab] OR "cpg" OR "epigenetic*"[tiab] OR "epigenom*"[tiab])

AND

("diabetes mellitus"[mh] OR "Insulin Resistance"[mh] OR "glucose"[mh] OR "insulin"[mh] OR "hyperinsulinism"[mh] OR "gestational diabetes"[tiab] OR "gestational diabetes mellitus"[tiab] OR "GDM"[tiab])

AND

("pregnancy" OR "pregnant" OR "gestational" OR "prenatal" OR "antenatal"))

Filter: English, humans

PubMed publisher adult type 2 diabetes associated CpGs:

(("Epigenomics"[mh] OR "Epigenesis, Genetic"[mh] OR "DNA methylation"[mh] OR "CpG Islands"[mh] OR "methylat*"[tiab] OR "cpg" OR "epigenetic*"[tiab] OR "epigenom*"[tiab]) AND

("diabetes mellitus"[mh] OR "Insulin Resistance"[mh] OR "glucose"[mh] OR "insulin"[mh] OR "hyperinsulinism"[mh])

AND

("level*"[tiab] OR "blood" OR "serum" OR "plasma*"[tiab] OR "concentration*"[tiab]))

Filter: English, humans
